# Supplementary material for: Single-Cell Transcriptome Analysis of Radiation Pneumonitis Mice
Source: Antioxidants (Basel). 2022 Jul 26;11(8):1457. doi: 10.3390/antiox11081457 (PMC9331247; doi:10.3390/antiox11081457)
Supplement: Supplementary file 1 [file antioxidants-11-01457-s001.zip › antioxidants-1782468-supplementary/supplementary Table S1.pdf]

Table S1: Differential cell proportions between all clusters in single-cell sequencing.

Table S1-1. Cell types identified and their marker genes.

Cell type	Marker genes	Ref
B cell	Ms4a1, Cd79a	1,2
T cell	Cd2, Cd3d, Trbc1	2
NK cell	Nkg7, Klrd1, Klrb1	3
Mixed lymphocyte	Ms4a1, Cd3d, Nkg7, Jchain	1,2
Plasma cell	Jchain, Mzb1	4
Macrophage	Mrc1, Cd68	3
Monocyte	Csf1r, Ifitm3, Ifitm2	5
Dendritic cell (DC)	Cd209a, Clec9a	6,7
Neutrophil	Csf3r ,Cxcr2	8
Erythroblast	Alas2, Hba-a2, Gypa	9
Alveolar type II cell (AT2)	Epcam, Sftpb, Sftpc	3
Fibroblast (Fib)	Dcn, Col1a1, Col3a1	2,4
Smooth muscle cell (SMC)	Acta2, Tagln, Tpm2	2
Club cell	Scgb1a1, Scgb3a2, Wfdc2	1,3,4
Endothelial cell (EC)	Cdh5, Pecam1, Cldn5	2

Table S1-2. The number of cells in different samples.

Cell type	Ctrl-57	53D	Ctrl-57B	53BD
T_cells	1798	2313	1996	5767
Macrophages	1497	3676	0	0
B_cells	811	1697	2382	7347
Monocytes	348	1462	453	1515
NK_cells	580	621	86	763
AT2	727	784	0	0
DCs	404	1087	0	22
Mixed_lymphocytes	529	337	133	585
Neutrophils	177	360	211	949
Fib	119	230	0	0
Club_cells	54	245	1	0
Plasma_cells	74	223	1	1
Erythroblasts	154	32	4	100
VEC	104	184	0	0
SMC	8	111	0	0

Table S1-3. The proportion of cells in different samples.

Cell type	Ctrl-57	53D	Ctrl-57B	53BD
T_cells	24.35%	17.31%	37.90%	33.83%
Macrophages	20.27%	27.51%	0.00%	0.00%
B_cells	10.98%	12.70%	45.22%	43.09%
Monocytes	4.71%	10.94%	8.60%	8.89%
NK_cells	7.85%	4.65%	1.63%	4.48%
AT2	9.85%	5.87%	0.00%	0.00%
DCs	5.47%	8.14%	0.00%	0.13%
Mixed_lymphocytes	7.16%	2.52%	2.53%	3.43%
Neutrophils	2.40%	2.69%	4.01%	5.57%
Fib	1.61%	1.72%	0.00%	0.00%
Club_cells	0.73%	1.83%	0.02%	0.00%
Plasma_cells	1.00%	1.67%	0.02%	0.01%
Erythroblasts	2.09%	0.24%	0.08%	0.59%
VEC	1.41%	1.38%	0.00%	0.00%
SMC	0.11%	0.83%	0.00%	0.00%
