# Supplementary figures and images for: Single-Cell Transcriptome Analysis of Radiation Pneumonitis Mice
Source: Antioxidants (Basel). 2022 Jul 26;11(8):1457. doi: 10.3390/antiox11081457 (PMC9331247; doi:10.3390/antiox11081457)

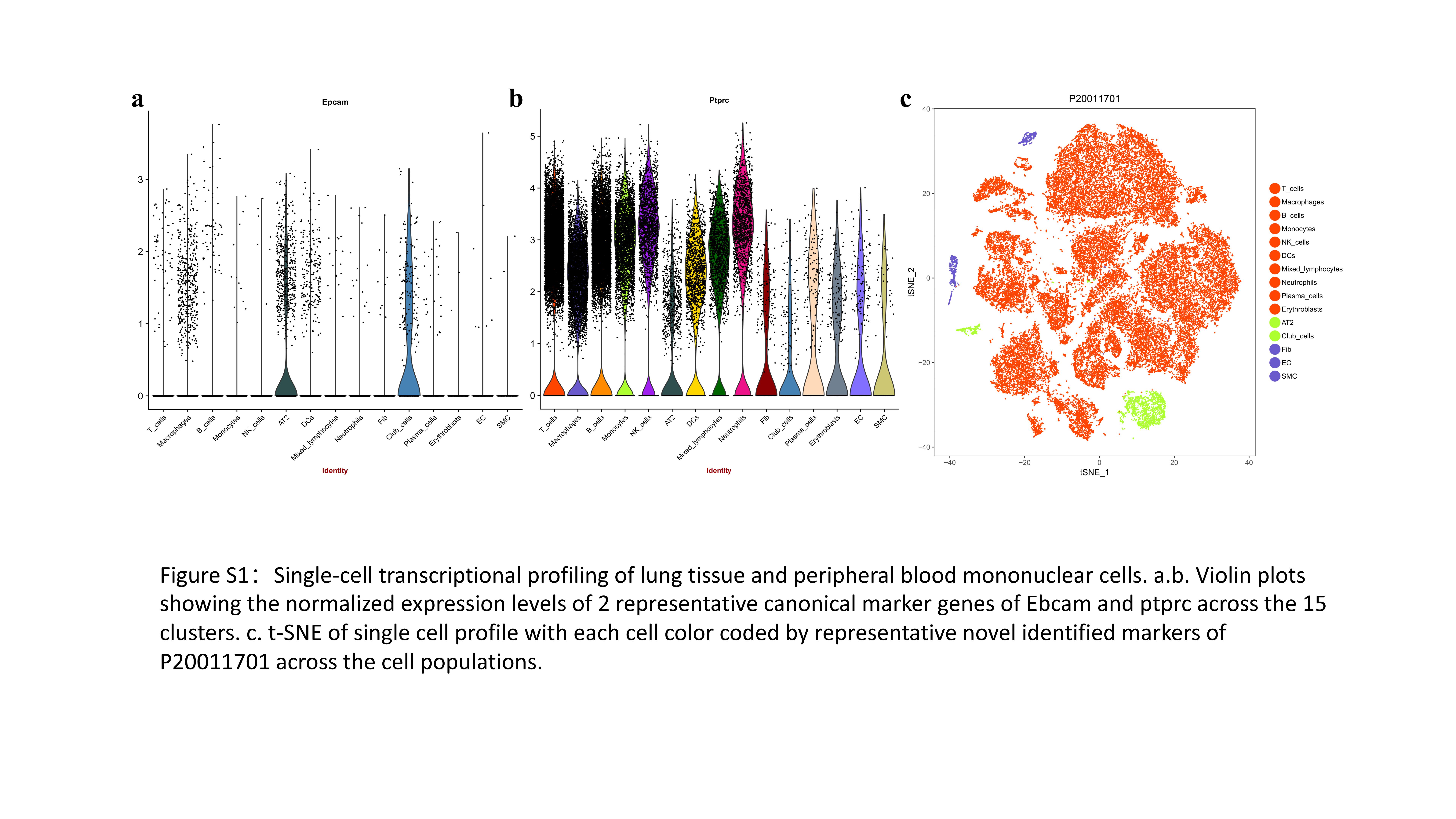

Supplement: Supplementary file 1 [file antioxidants-11-01457-s001.zip › antioxidants-1782468-supplementary/Figure S1.jpg]

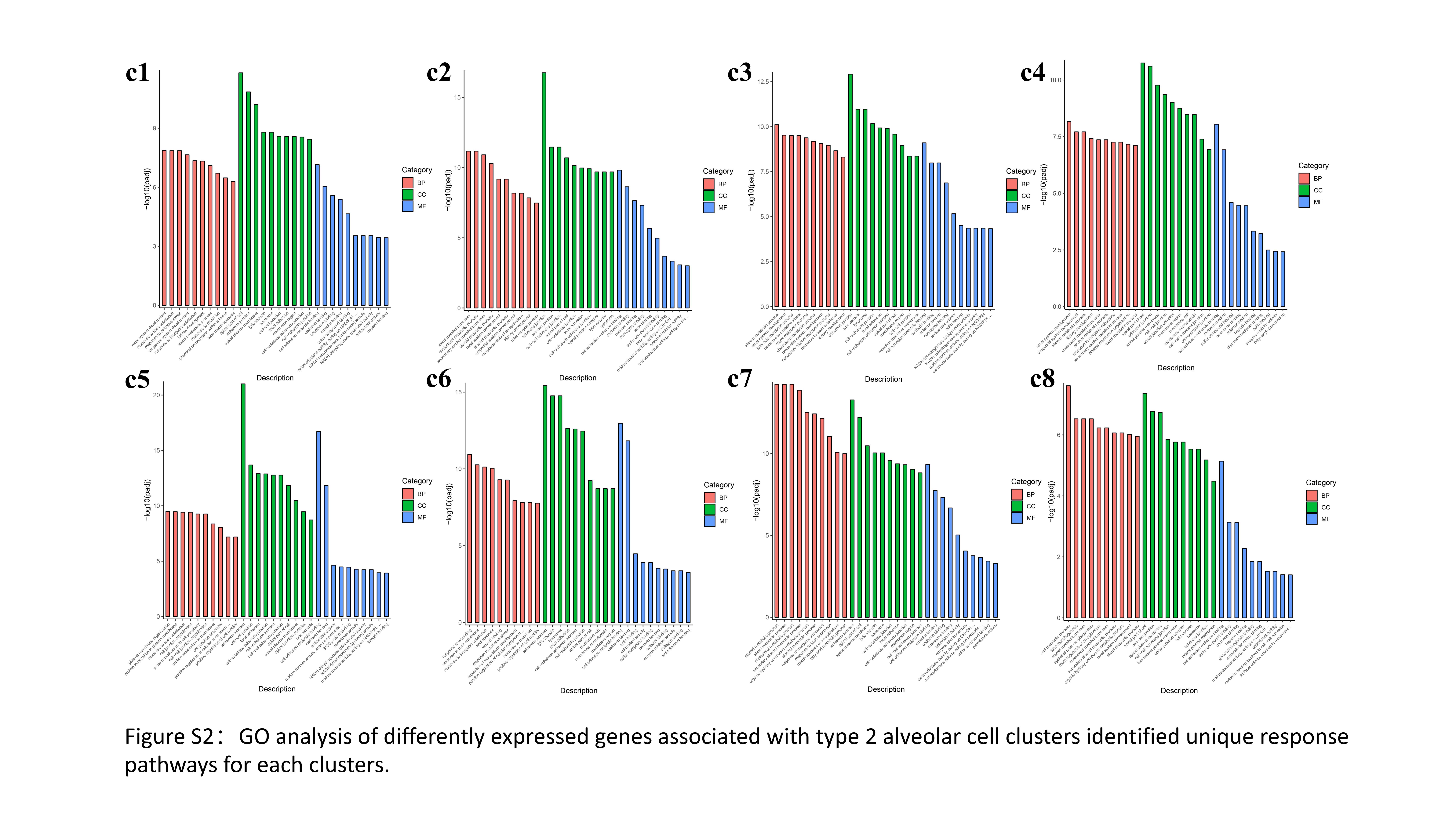

Supplement: Supplementary file 1 [file antioxidants-11-01457-s001.zip › antioxidants-1782468-supplementary/Figure S2.jpg]

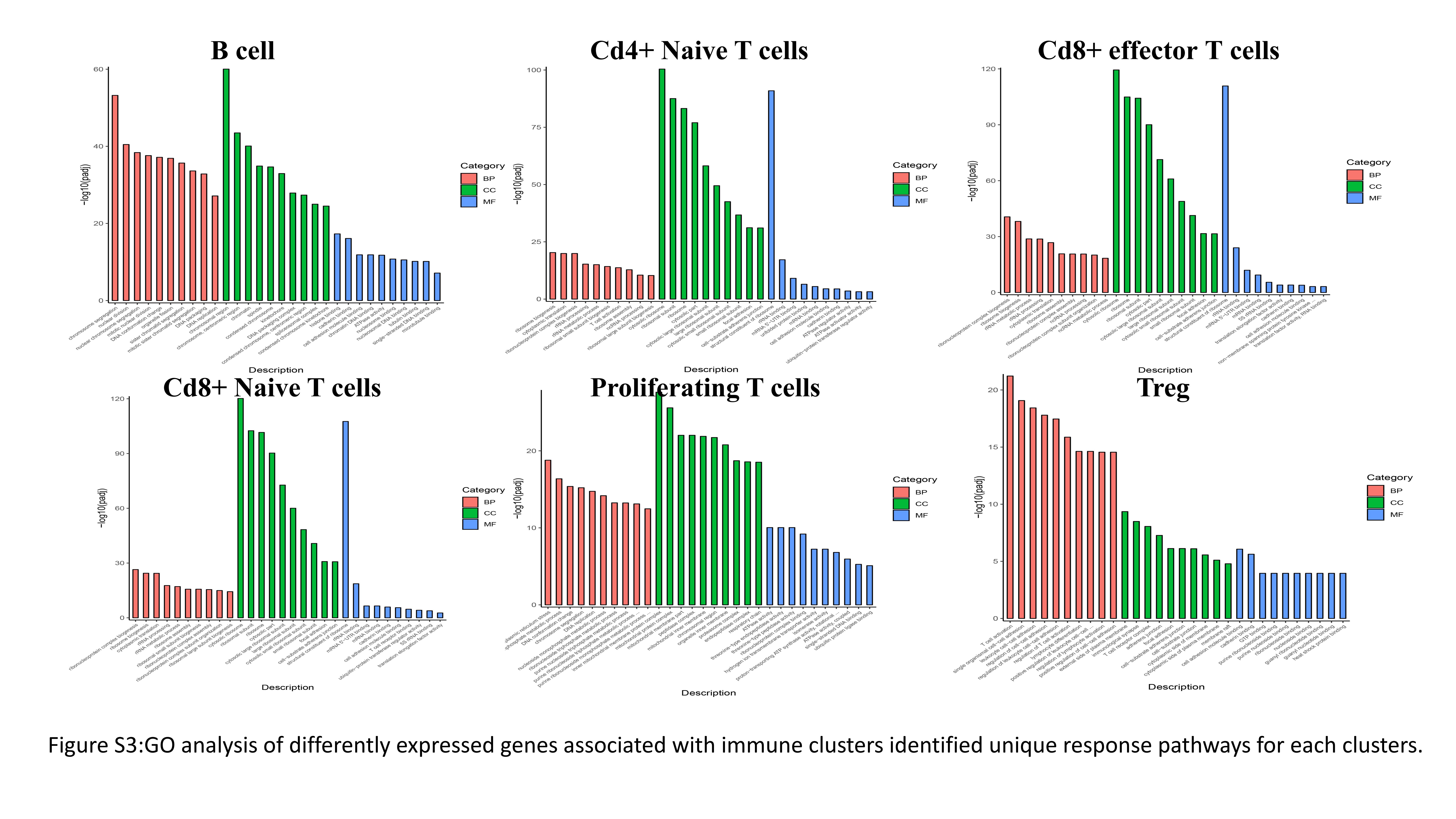

Supplement: Supplementary file 1 [file antioxidants-11-01457-s001.zip › antioxidants-1782468-supplementary/Figure S3.jpg]

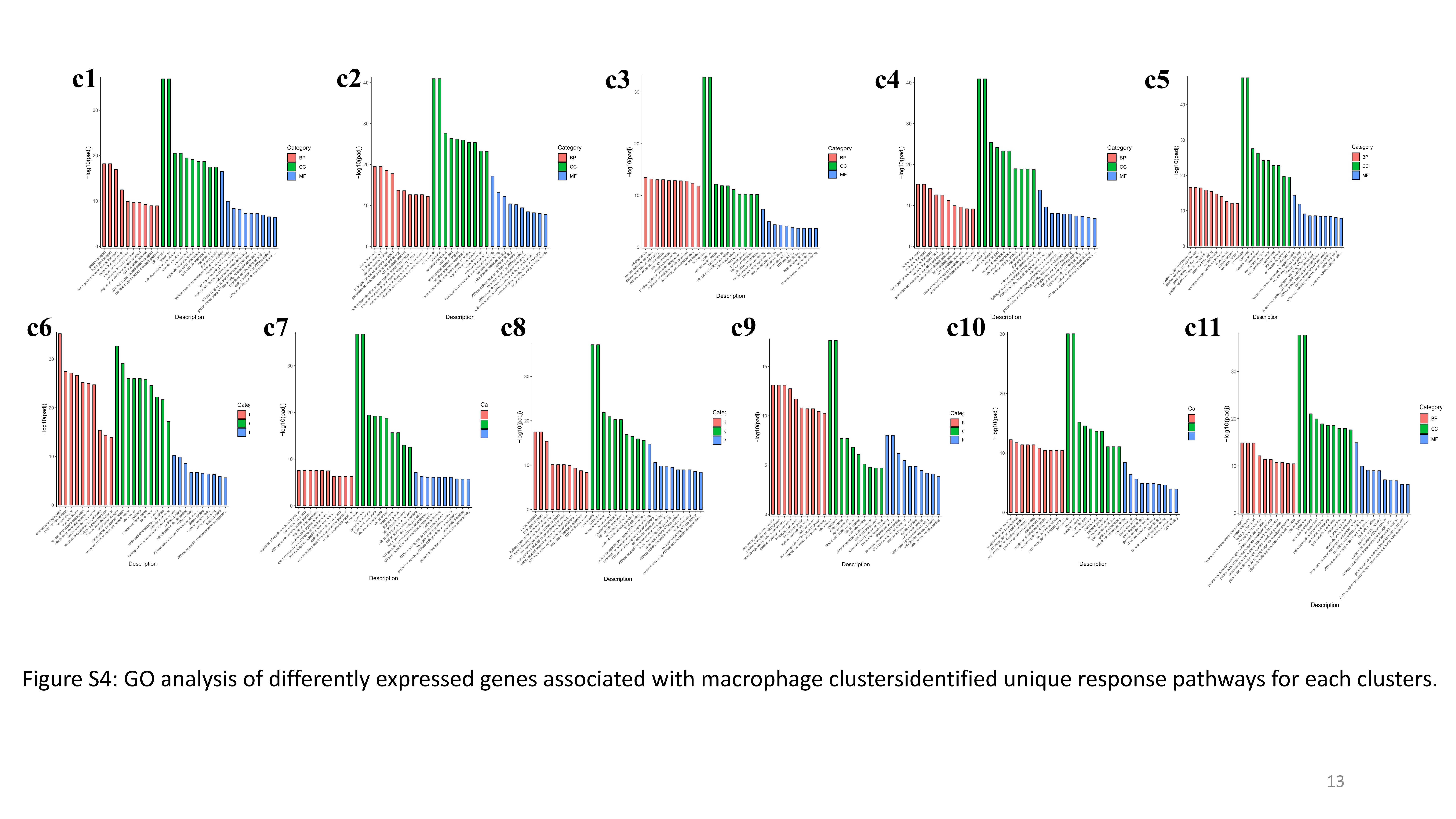

Supplement: Supplementary file 1 [file antioxidants-11-01457-s001.zip › antioxidants-1782468-supplementary/Figure S4.jpg]
